# 323. Outpatient Initiation and Successful Completion of Intravenous Antibiotic Therapy at Home for Eligible Patients: A Quality Improvement Project

**DOI:** 10.1093/ofid/ofad500.394

**Published:** 2023-11-27

**Authors:** Mark Henry Cedeno, Ruth Serrano, Lauren Flores, Jason Bowling, Heta Javeri

**Affiliations:** UT Health San Antonio, San Antonio, Texas; UT Health San Antonio, San Antonio, Texas; UT Health San Antonio, San Antonio, Texas; UT Health San Antonio, San Antonio, Texas; UT Health San Antonio, San Antonio, Texas

## Abstract

**Background:**

Outpatient Parenteral Antibiotic Therapy (OPAT) is known to reduce hospital length of stay, readmissions, hospital morbidity, increase patient satisfaction, and reduce costs. Initiating IV antibiotics directly in the outpatient setting requires coordination with a multidisciplinary team and can be a lengthy process. Outpatient initiations can prevent hospitalizations and unnecessary ED visits for eligible patients. This strategy became critical during the COVID-19 pandemic given limited hospital capacity. The aim of this project was to streamline the process of initiating IV antibiotics in the outpatient setting for eligible patients and reduce time-to-initiation to less than seven days in our institution.

**Methods:**

We mapped the steps for outpatient initiations and identified factors leading to delays. Through a prospective and retrospective chart review, we identified TTI, hospitalizations prevented, diagnosis (figure 1), and 60-day relapse. TTI was represented in an SPC chart, highlighting the variability in days among patients. We created an action plan to improve the processes of obtaining insurance approval, establishing IV access, and a standardized protocol. Return on investment was calculated.

**Results:**

We coordinated outpatient initiations for 31 patients from Dec 2020 to Mar 2023. TTI varied (4-21 days, median 11 days) (figure 2). There was no difference in TTI of the Dalbavancin group vs non-dalbavancin group (median 10 and 10.5 days, respectively). After implementing a standardized protocol in Dec 2022, three patients were started on IV antibiotics. Although the protocol outlined steps and improved communication, the TTI remained similar (4-23 days, median 10.5 days). Delays were driven by insurance approval. Two patients were started within the goal of seven days. Of 31 outpatient starts, one patient had a 60-day relapse. We calculated savings between $102,000 to $168,000, driven by ED and hospital admission diversion.

**Figure 1:**
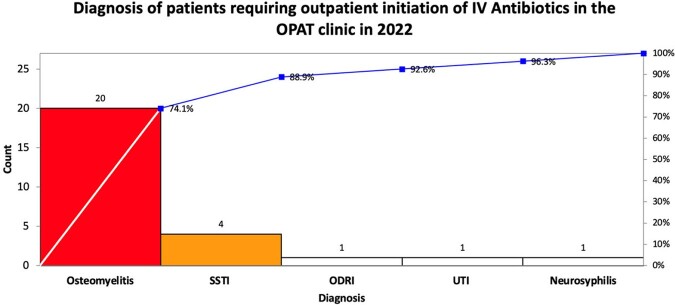
Common diagnoses for eligible OPAT initiation

Pareto chart depicting the number and frequency of diagnoses in which IV antibiotics were initiated from our OPAT clinic. SSTI, skin/soft tissue infection; ODRI, orthopedic device-related infection; UTI, urinary tract infection.

**Figure 2:**
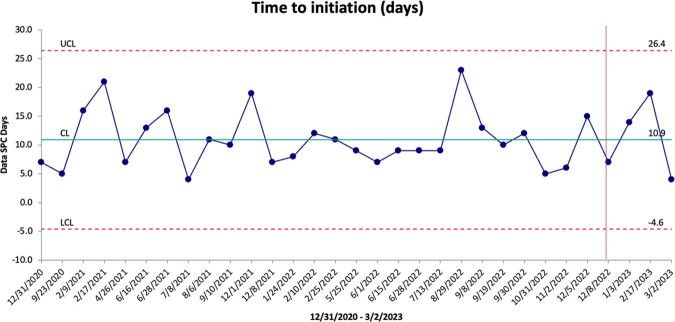
Time-to-initiation (TTI) in days

Statistical process control (SPC) chart depicting the time-to-initiation in days for each subject started on IV antibiotics from our OPAT clinic. The vertical line indicates the date we implemented our standardized protocol for outpatient initiations.

**Conclusion:**

Despite not meeting our goal, we made strides in implementing a standardized protocol that streamlines communication to reduce delays in a cost-effective manner while improving patient satisfaction, continuity of care, and resource utilization. This model can help implement more efficient protocols of this kind nationwide.

**Disclosures:**

**Jason Bowling, MD**, Eli Lilly: Advisor/Consultant

